# Discovery of a bacterial peptide as a modulator of GLP-1 and metabolic disease

**DOI:** 10.1038/s41598-020-61112-0

**Published:** 2020-03-18

**Authors:** Catherine Tomaro-Duchesneau, Stephanie L. LeValley, Daniel Roeth, Liang Sun, Frank T. Horrigan, Markus Kalkum, Joseph M. Hyser, Robert A. Britton

**Affiliations:** 1 Department of Molecular Virology and Microbiology, Baylor College of Medicine, Houston, Texas United States of America; 2 Department of Molecular Imaging and Therapy, Beckman Research Institute of the City of Hope, Duarte, California, United States of America; 3 Department of Molecular Physiology and Biophysics, Baylor College of Medicine, Houston, Texas United States of America

**Keywords:** Microbiology, Metabolism

## Abstract

Early work in rodents highlighted the gut microbiota’s importance in metabolic disease, including Type II Diabetes Mellitus (T2DM) and obesity. Glucagon-like peptide-1 (GLP-1), an incretin secreted by L-cells lining the gastrointestinal epithelium, has important functions: promoting insulin secretion, insulin sensitivity, and β-cell mass, while inhibiting gastric emptying and appetite. We set out to identify microbial strains with GLP-1 stimulatory activity as potential metabolic disease therapeutics. Over 1500 human-derived strains were isolated from healthy individuals and screened for GLP-1 modulation by incubating bacterial cell-free supernatants with NCI H716 L-cells. Approximately 45 strains capable of increasing GLP-1 were discovered. All GLP-1 positive strains were identified as *Staphylococcus epidermidis* by 16S rRNA sequencing. Mass spectrometry analysis identified a 3 kDa peptide, Hld (delta-toxin), present in GLP-1 positive supernatants but absent in GLP-1 neutral supernatants. Studies in NCI-H716 cells and human jejunal enteroids engineered to make more enteroendocrine cells demonstrated that Hld alone is sufficient to enhance GLP-1 secretion. When administered in high-fat-fed mice, Hld-producing *S. epidermidis* significantly reduced markers associated with obesity and T2DM. Further characterization of Hld suggests GLP-1 stimulatory action of Hld occurs via calcium signaling. The presented results identify a novel host-microbe interaction which may ultimately lead to the development of a microbial peptide-based therapeutic for metabolic disease.

## Introduction

Metabolic disorders, including Type 2 Diabetes Mellitus (T2DM) and obesity, pose a serious public health concern both nationally and globally. According to the Centers for Disease Control and Prevention (CDC), 9.4% of the population of the United States has Diabetes, including 25.2% of those aged 65 years or older^1^. Obesity is also a major health concern, with more than one-third of American adults considered obese^2^. Current treatment approaches, including intensive lifestyle modifications, diet intervention, and pharmacologics, have proven unsuccessful in controlling the global increase of metabolic disorders. Therefore, a novel approach to combat metabolic disorders is needed.

A number of research groups have recently demonstrated the role of the human gut microbiota in metabolism and metabolic disease, leading to the attempt to develop microbial therapeutics. Bäckhed *et al*. initially spearheaded this research; using germ-free rodents, it was demonstrated that when the microbiota of conventionally raised animals was transplanted into germ-free rodents, the latter developed an increase in adiposity and insulin resistance^3^. Studies by a number of different groups also demonstrated the importance of the microbiota in metabolism, described in a recent review^4^. Specifically, the gut microbiota has been shown to play an important role in gut hormone modulation, including GLP-1. Administration of prebiotics, non-digestible food ingredients that stimulate the growth of specific organisms of the microbiota, increased GLP-1 concentrations, correlating with appetite, fat mass, and hepatic insulin resistance^5^. Samuel *et al*. demonstrated that n-butyrate, a short-chain fatty acid originating from the gut microbiota, increased GLP-1 production^6^. As well, Yadav *et al*. demonstrated an increase in GLP-1 levels in mice following administration of a butyrate-producing probiotic^7^. Everard *et al*. showed that the abundance of *Akkermansia muciniphila* is correlated with increased intestinal levels of 2-oleoylglycerol (2-OG), which stimulates GLP-1 secretion from intestinal L cells in type 2 diabetic mice^8^. Specifically, 2-OG has been shown to be an agonist of GPR119, a receptor that plays a key role in promoting GLP-1 release in humans. Although promising results have been observed for microbial therapeutics, a successful therapeutic capable of combatting metabolic disorders, particularly T2DM and obesity, has yet to be developed. Moreover, the exact role of the microbiota on GLP-1 modulation remains to be elucidated and investigated as a therapeutic target. The present work aimed to identify human-derived bacterial strains capable of stimulating GLP-1 secretion, with the goal of developing a metabolic disease therapeutic.

## Methods

### Bacterial strain isolation

Strains JA1, JB1, JD11, and JA8 were isolated from human breast milk from a female who had been lactating for four months. Prior to collection, the surface of the areola was sterilized with 70% (v/v) ethanol wipes. Milk was collected using a freshly-sterilized adapter and bottle. After collecting 1 mL, the collection bottle was replaced with a new, sterile bottle and collection continued until natural cessation of milk flow. This latter volume was used for isolation. Bacteria were concentrated from breast milk by centrifugation at 1789 × g for 10 min, resuspended in a small volume of supernatant (whey fraction), and spread plate onto Brain Heart Infusion (Difco) supplemented with yeast extract (BHIS) (JA1, JB1, JA8) or Hyp1 medium (JD11) plates and incubated at 37 °C in a hypoxic chamber with atmosphere of 2% O_2_, 5% CO_2_, 93% N_2_. Individual colonies were re-streaked twice on the same agar medium (BHIS or Hyp1) to ensure homogeneity. The majority of colonies from the second re-streaked plate were scraped into liquid medium amended with 15% (v/v) glycerol and stored at −80 °C. All other strains screened in this study were isolated from healthy human volunteer fecal samples or intestinal biopsies. All samples were collected under approved protocols by the Institutional Review Board of Michigan State University. All volunteers were over the age of 18 and samples were collected under informed consent that was obtained from each subject. No identifiable information was collected during sample procurement.

### rRNA sequencing of isolates

To identify the bacterial isolates, bacteria were streaked on GM17 agar plates from frozen stock and incubated at 37 °C for 1–2 days. Bacterial colony mass was then resuspended in 100 µL of water and transferred to sterile bead beating tubes and homogenized for 2 min in a mini-beadbeater-96 (Biospec Products). Tubes were centrifuged at 8000 × g for 30 sec and supernatants were used for 16S rRNA gene PCR amplification. The final 25 µL PCR reactions contained 1 µL of template, 1X Phusion, High Fidelity Buffer (New England Biolabs), 200 µM dNTPs (Promega), 10 nM primers (8 F and 1492 R) and 0.225 units of Phusion DNA Polymerase (New England Biolabs). The amplification cycle consisted of an initial denaturation at 98 °C for 30 sec, followed by 26 cycles of 10 sec at 98 °C, 20 sec at 51 °C, and 1 min at 72 °C. Amplification was verified by agarose gel electrophoresis. For sample cleanup, 1 µL of Exo-SAP-IT (ThermoFisher) was added to 2.5 µL of PCR product and incubated at 37 °C for 15 min followed by a 15 min incubation at 80 °C to inactivate the enzyme. The product was cooled, and 5.5 µL of water and 1 µL of 10 µM 1492 R primer were added and sent to Genewiz for sequencing.

### Bacterial growth and preparation of cell-free supernatants

Bacterial isolates were streaked from frozen glycerol stocks onto GM17 agar plates and incubated anaerobically overnight at 37 °C. One colony was inoculated into 5 mL of GM17 broth and incubated overnight at 37 °C followed by one more subculture into GM17 broth, and incubation overnight at 37 °C. Once grown, bacterial cultures were centrifuged at 5000 × g for 20 min. Supernatants were collected and lyophilized (Labconco Freezone), followed by storage at −80 °C until used for subsequent assays. For size fractionation studies, bacterial cell-free supernatants were separated by size using centrifugal filter units (Amicon).

### Screening for GLP-1 stimulatory activity using *in vitro* enteroendocrine cell models

NCI H716 (American Type Culture Collection (ATCC) CCL-251) cells were grown in Roswell Park Memorial Institute (RPMI, ATCC) medium supplemented with 10% (v/v) heat inactivated newborn calf serum (NBCS). Cultures were maintained at a concentration of 2–8 × 10^5^ cells/mL and used at passages 15–40 for cell studies. For cell studies, 96-well plates were coated with 100 µL of 10 mg/mL Matrigel (BD Biosciences) for 2 h at room temperature. Following coating, NCI H716 cells were seeded at a concentration of 1 × 10^5^ cells/well in Dulbecco’s Modified Eagle’s Medium (DMEM) supplemented with 10% (v/v) NBCS, as determined by trypan blue staining using a hemocytometer. Two days later, lyophilized bacterial supernatants, including GM17 as a negative control, were resuspended in Krebs buffer containing bovine serum albumin (BSA, 0.2% w/v) and bovine bile (0.03% w/v) and incubated on the NCI H716 cells at 37 °C with 5% CO_2_. 4-phorbol 12-myristate, 13-acetate (PMA, 2 µM) was used as a positive control as it is a potent stimulator of GLP-1 secretion through activation of protein kinase C (PKC). Following a 2 h incubation, supernatants were collected and analyzed for GLP-1 levels by ELISA (Millipore Sigma) according to the manufacturer’s protocol. Cell viability was monitored using PrestoBlue Cell Viability Reagent (ThermoFisher Scientific) following the manufacturer’s instructions. GLUTag cells were generously gifted by Dr. Colin Leech (The State University of New York Upstate Medical University). GLUTag cell experiments were performed following the same protocol as for NCI H716 cells but with seeding of the cells directly into the 96-well plates, with no need for Matrigel coating due to their adherent nature.

### Mouse studies

To investigate whether a GLP-1 stimulating bacterial strain identified *in vitro* could have an effect on metabolic disease markers *in vivo*, we performed a mouse study. We used 8 week old female C57BL/6 humanized microbiota mice established by Collins *et al*.^9^. Mice were housed three per cage in a room with controlled temperature, humidity, and alternating light and dark cycle (12:12 h light/dark cycle). The two diets were obtained from Research Diets (New Jersey, USA): high fat diet (D12492) containing 60 kcal% fat and control diet (D12450B) containing 10 kcal% fat. Mice were randomized by mass into three groups (n = 6): 1) normal fat diet treated with vehicle (GM17 culture media), 2) high fat diet treated with vehicle, and 3) high fat diet treated with 2 × 10^8^ cells/mouse *S. epidermidis* JA1 culture from freshly grown overnight cultures. Mice were allowed free access to food and water. The experiment lasted for 16 weeks with treatments administered five times a week by intragastric gavage. Food intake and body mass were monitored twice a week. Serum was collected every two weeks in fasted animals (6 hours without food) by venous tail bleed for glucose and insulin measurements. Oral glucose tolerance tests (2 g glucose/kg animal mass) were performed every four weeks. Mice were euthanized after a 6 h fast by carbon dioxide asphyxiation and blood was drawn by cardiac puncture. Gonadal adipose mass was dissected and massed as a marker of adiposity. Experimental protocols were reviewed and approved by the Institutional and Animal Care Use Committee (IACUC) of Baylor College of Medicine. All experimental procedures were carried out according to the approved protocol and guidelines set by the IACUC.

### Mass spectrometry analysis of bacterial supernatants

To identify the bacterial compound responsible for GLP-1 stimulation, we performed mass spectrometry analysis. Lyophilized bacterial supernatants collected from overnight cultures of two GLP-1 positive strains (JA1 and JA8) and two neutral strains (JB1 and JD11) were reconstituted in 50 µL water. Proteins were denatured by the addition of trifluoroethanol (50 µL), reduced with tris(2-carboxyethyl)phosphine, and alkylated with iodoacetamide. Samples were diluted with 900 µL ammonium bicarbonate buffer (100 mM) and trypsin/LysC was added. The next day, samples were acidified with formic acid and analyzed on an Orbitrap Fusion mass spectrometer equipped with an Easy nanospray HPLC system with a PepMap RSLC C18 column (Thermo Fisher Scientific). Protein identification and relative-quantification by spectra counting were done using Proteome Discoverer 2.0 (Thermo Fisher Scientific) and Scaffold 4 (Proteome software) using a 1% false discovery rate on the protein and peptide level.

### Peptide exposure on NCI H716 cells

To investigate whether the Hld peptide from *S. epidermidis* (Hld_Se_) identified by mass spectrometry recapitulates the GLP-1 stimulatory activity seen with the bacterial supernatants, the Hld_Se_ peptide was synthesized as well as the *S. aureus* Hld (Hld_Sa_) and the mutant peptides 24_25insT and A3Q. Hld_Se_ (MAADIISTIGDLVKWIIDTVNKFKK), Hld_Sa_ (MAQDIISTIGDLVKWIIDTVNKFTKK), Hld_Se_ 24_25insT (MAADIISTIGDLVKWIIDTVNKFTKK) and Hld_Se_ A3Q (MAQDIISTIGDLVKWIIDTVNKFKK) were synthesized by LifeTein (New Jersey, USA) at 98% purity with an f-Met modified N-terminus. NCI H716 cell monolayers were prepared as previously described^10^. The four peptides were suspended in Krebs buffer containing 0.2% w/v BSA and 0.03% w/v bovine bile, at various concentrations to obtain a dose response curve, and incubated on the NCI H716 cells for 2 h. GLP-1 levels and cell viability were monitored, as previously described.

### Calcium signaling in HEK293-GCAMP6s cells

HEK293 were transduced with a lentivirus encoding the GCaMP6s calcium sensor (HEK293-GCaMP6s) and a stable cell line was selected using 5 μg/mL puromycin treatment, as previously described^11^. For calcium imaging cells were plated into Greiner Bio-One^TM^ CELLSTAR μClear flat bottomed black 96-well plates that were coated with poly-D-lysine. Calcium responses to Hld_Sa_ or Hld_Se_ were determined using time-lapse fluorescence microscopy by widefield epifluorescence imaging using a Nikon TiE inverted microscope. Cells were imaged with widefield epifluorescence using a 20x PlanFluor (NA 0.45) phase contrast objective, using a SPECTRA X LED light source (Lumencor) for green fluorescence. Images were acquired with a 100 ms exposure and a 2 sec interval between acquisitions. Images were recorded using an ORCA-Flash 4.0 sCMOS camera (Hamamatsu) and Nikon Elements v4.5 software was used for data acquisition and image analysis. Cell were washed and placed in normal Ringer’s buffer (160 mM NaCl, 4.5 mM KCl, 2 mM CaCl_2_, 1 mM MgCl_2_, 10 mM HEPES, pH 7.4) or Low Ca^2+^ Ringer’s where CaCl_2_ was omitted and 1 mM Ethylenediaminetetraacetic acid (EDTA) added. To determine the cytosolic Ca^2+^ response to Hld_Sa_ or Hld_Se_, baseline fluorescence was measured for 30 seconds and then cells were treated with 5 μM peptide and imaged for 3.5 minutes. The cytosolic Ca^2+^ response was determined as the change in GCaMP6s fluorescence (∆FGCaMP6s) from baseline to the maximum post-treatment value and the GCaMP6s area under the curve (AUC) was determined using GraphPad Prisim software.

### Patch clamping of NCI H716 cells

Membrane potentials were recorded using the current-clamp mode in the whole-cell configuration. The pipette solution contained 130 mM KOH, 5 mM KCl, 5 mM NaCl, 1 mM MgCl_2_, 10 mM HEPES, and 1 mM EGTA. The pH was adjusted to 7.2 with MES. The bath solution contained 5 mM KCl, 135 mM NaCl, 2 mM CaCl_2_, 1 mM MgCl_2_, and 10 mM HEPES. The pH was adjusted to 7.2 with NaOH. 5 µM of Hld_Se_ or Hld_Sa_ were added to the bath solution with a Perfusion Fast-Step (SF-77B) system. Experiments were performed at room temperature (20–22 °C). Data were acquired with an Axopatch 200B amplifier (Axon Instruments Inc.) with the Axopatch’s filter set at 100 kHz. Signals were subsequently filtered by an 8-pole Bessel filter (Frequency Device Inc.) at 5 kHz and sampled at 200 kHz with an 18-bit A/D converter (Instrutech ITC-18).

### NGN3-Human intestinal enteroids (HIE)

We recently developed a novel human intestinal enteroid model of enteroendocrine cells using overexpression of the transcription factor neurogenin-3 (NGN3-HIE)^12^. We used the NGN3-HIE model in this work to investigate whether Hld_Se_ can stimulate the release of other enteroendocrine cell molecules. We laid down flat NGN3-HIE monolayers, following the previously described protocol^12^ . Hld_Se_ was suspended in Krebs buffer containing 0.2% w/v BSA and 0.03% w/v bovine bile, at 20 and 40 µM and incubated on the NGN3-HIE monolayers for 2 h. Cell viability was monitored by PrestoBlue, as previously described. A Milliplex Multiplex assay was performed using a Luminex kit (Millipore Sigma) to measure GLP-1, glucagon, PYY, and GIP, according to the protocol provided by the manufacturer. Serotonin secretion was quantified by ELISA (Eagle Biosciences) according to the manufacturer’s instructions.

### Statistical analysis

Statistical analyses were performed using GraphPad Prism version 7.0 (San Diego, CA, USA). Experimental results are expressed as means ± standard deviation. Statistical significance was set at p < 0.05. One-way statistical comparisons were carried out using one-way analysis of variance (ANOVA), followed by multiple comparisons of the means using Tukey’s post-hoc analysis for the GLP-1 secretion experiments in NCI H716 cells, gonadal adipose mass, fasted insulin levels, membrane potential and luminex data with the enteroids. Statistical comparisons for calcium flux assays were carried out by Kruskal-Wallis One-way ANOVA because the data was not normally distributed. Two-way ANOVA analysis was performed for animal mass, food consumption, and the size fractionation experiments.

## Results

### Screening of a human-derived microbial library for regulation of the incretin hormone GLP-1

In order to identify bacterial strains capable of eliciting GLP-1 secretion, ~1500 microbial strains were screened using the GLP-1 secreting human cell line NCI H716^13^. Cell-free supernatants of each microbe were prepared and applied to monolayers of NCI-H716 cells for 2 hours. GLP-1 secreted into the medium was analyzed by ELISA. Of the 1500 strains that were screened, the vast majority (>1400) had no influence on GLP-1 secretion. We identified 45 isolates that showed increased GLP-1 secretion similar to or above stimulation of GLP-1 with the positive control phorbol 12-myristate 13-acetate (PMA). We also identified 25 strains that dramatically reduced the level of secreted GLP-1; these strains were not further characterized as part of this study^14^.

**Figure 1:**
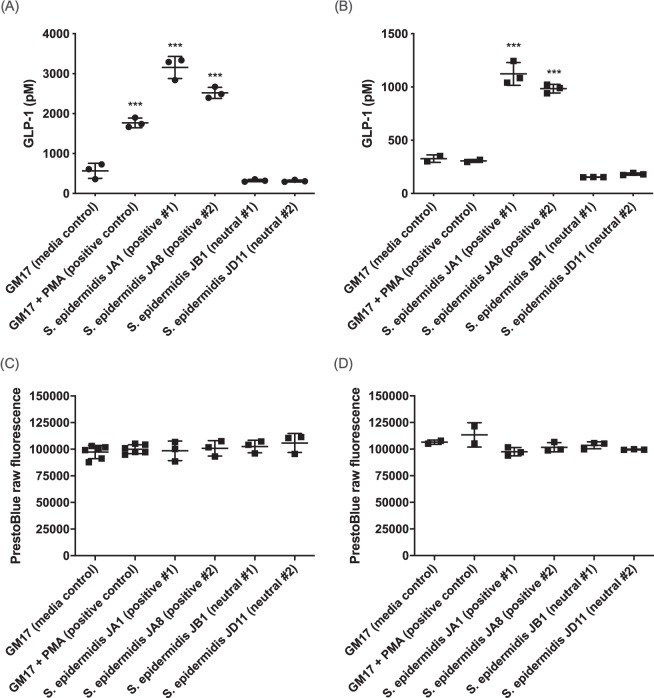
Glucagon-like peptide-1 levels are stimulated following exposure to cell-free *S. epidermidis* supernatants for 2 hours using **(A)** NCI H716 and **(B)** GLUTag cells, measured by ELISA. *S. epidermidis* supernatants did not impact the cell viability of **(C)** NCI H716 and **(D)** GLUTag cells, measured using a resazurin-based PrestoBlue assay. (***p < 0.001 by One-way ANOVA). The GM17 medium control is lyophilized GM17 resuspended in Krebs buffer. Each point on the graph represents the average of three biological replicates of the respective experiments.

16S rRNA sequencing of all 45 stimulatory strains identified them as *S. epidermidis* isolates. We originally isolated most of these strains from either breast milk or fecal samples from healthy human volunteers. To further characterize the impact of *S. epidermidis* strains on GLP-1 secretion, we incubated cell-free supernatants from two of the stimulatory strains with the highest activity, *S. epidermidis* JA1 and JA8, with NCI H716 cells. JA1 and JA8 stimulated a release of 3155 ± 276 pM and 2518 ± 141 pM GLP-1, respectively (Fig. 1A). The GM17 media control and the PMA positive control had GLP-1 levels of 565 ± 188 pM and 1767 ± 120 pM GLP-1, respectively, indicating a 5–6 fold increase in activity by JA1 and JA8 over the medium control.

To interrogate the robustness of the impact of *S. epidermidis* on GLP-1 secretion, we confirmed that JA1 and JA8 conditioned supernatant could stimulate GLP-1 secretion in a widely used GLP-1 secretion model, murine GLUTag cells^15^. The GM17 media control and the PMA positive control had GLP-1 levels of 327 ± 35 pM and 305 ± 14 pM, respectively, indicating no stimulation by PMA in this cell line. *S. epidermidis* JA1 and JA8 led to the release of 1123 ± 107 pM and 984 ± 40 pM GLP-1, respectively, indicating a 3–4 fold increase in GLP-1 secretion (Fig. 1B). These results support the role of a secreted factor in stimulating the release of GLP-1.

Interestingly, two strains (JB1 and JD11) of *S. epidermidis* in our library had no impact on the ability to stimulate GLP-1 secretion (denoted neutral), in both NCI H716 and GLUTag cells. None of the *S. epidermidis* bacterial cell-free supernatants had detectable toxicity on NCI H716 (Fig. 1C) and GLUTag (Fig. 1D) cells, as determined using PrestoBlue, a resazurin-based viability assay.

### *S. epidermidis* JA1 reduces markers of metabolic disease

**Figure 2:**
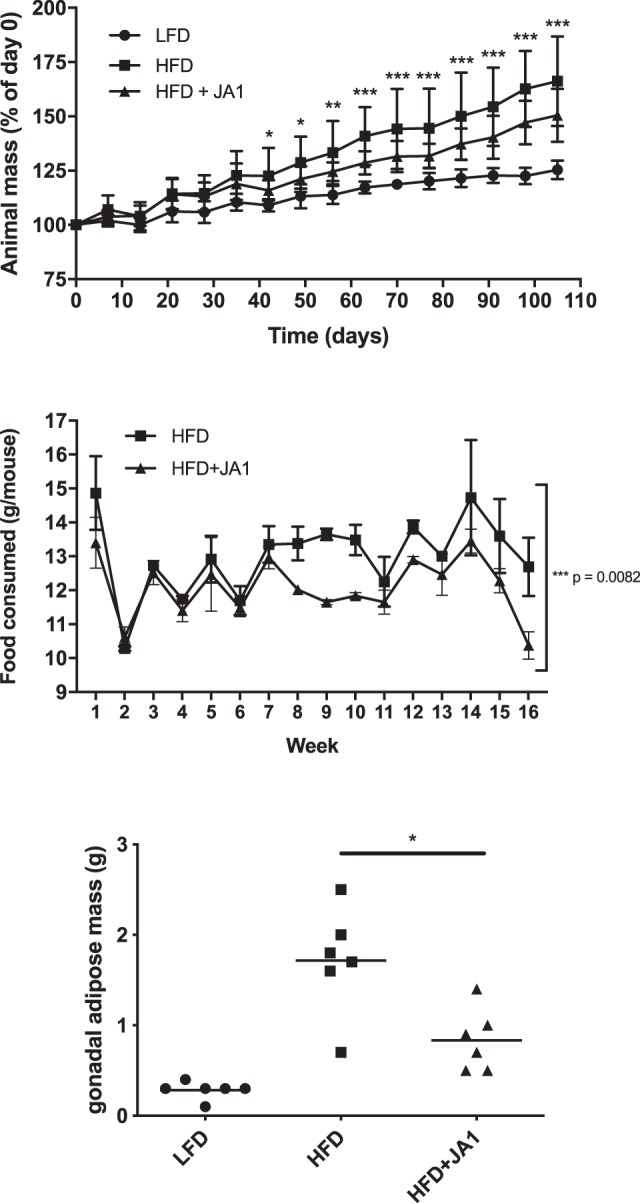
Impact of administration of *S. epidermidis* JA1 on (**A**) body weight and (**B**) food consumption throughout the 16 week study, and (**C**) gonadal adiposity at the end of the 16 week study in mice administered a high-fat diet. For (**A**), the * indicates the p-values of a two-way ANOVA analysis at the various timepoints (*p < 0.05, **p < 0.01, ***p < 0.001). For (**B**), the * indicates a significance of the time effect in a two-way ANOVA. For (**C**), the * indicates significance (p < 0.05) in a one-way ANOVA.

Following identification of JA1 as the strongest stimulator of GLP-1 secretion *in vitro*, we investigated its ability to modulate markers of metabolic disease during a 16-week study in a high-fat murine model. Mice were placed on a high-fat diet and gavaged with either *S. epidermidis* JA1 or GM17 medium as a negative control 5 times per week. Administration of *S. epidermidis* JA1 to HFD-fed mice for 16 weeks reduced markers of obesity, body mass, and adiposity compared to the GM17 medium control. Mice fed a HFD gained significantly more mass than mice on the conventional diet and administration of JA1 significantly reduced animal mass in mice fed a HFD (Fig. 2A). This significant difference was noted as of day 42 (*p* < 0.05), and at every time point for the rest of the study, with animal percent body mass of 122.6 ± 13.0% and 115.9 ± 4.7% for the HFD-fed mice and HFD-fed mice administered JA1, respectively.

**Figure 3:**
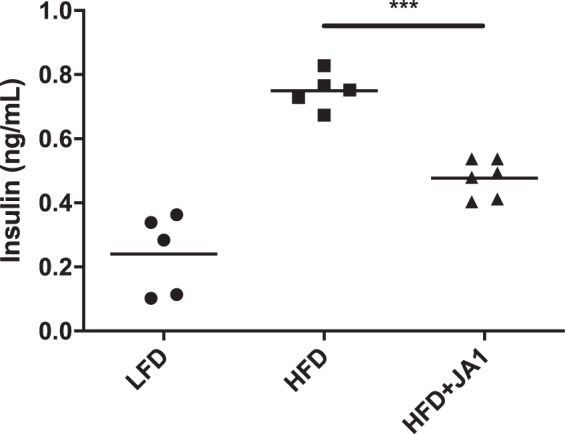
Impact of HFD and administration of *S. epidermidis* JA1 on fasted serum insulin levels at the end of the 16-week study (***p < 0.001, One-way ANOVA).

Since we hypothesized that *S. epidermidis* JA1 administration enhances the secretion of GLP-1, a satiety hormone, we also monitored food consumption throughout the 16-week study (Fig. 2B). The average food consumption for the HFD-fed mice was 13.0 ± 1.15 g/week compared to 12.09 ± 0.89 g/week for the HFD-fed mice administered *S. epidermidis* JA1 (*p* = 0.0082), demonstrating that *S. epidermidis* JA1 reduced food intake. To assess adiposity, the gonadal adipose tissue was measured at the end of the study (Fig. 2C). Mice fed a HFD had significantly more (*p* < 0.0001) adipose tissue mass (1.72 ± 0.59 g) than their LFD-fed counterparts (0.28 ± 0.10 g). Administration of *S. epidermidis* JA1 significantly reduced (*p* = 0.004) the levels of adipose tissue mass (0.83 ± 0.34 g). *S. epidermidis* JA1 administration in HFD-mice also reduced the levels of fasted hyperinsulinemia. Feeding with a HFD (0.75 ± 0.06 ng/mL) significantly elevated (*p* < 0.0001) the levels of fasted serum insulin as compared to mice administered the LFD (0.24 ± 0.12 ng/mL) (Fig. 3). Administration of *S. epidermidis* JA1 (0.48 ± 0.06 ng/mL) significantly reduced (*p* = 0.0004) fasted serum insulin levels in HFD-fed mice. Taken together, this data suggests potential for the modulation of metabolic markers by *S. epidermidis* JA1.

### Identifying the *S. epidermidis* compound responsible for GLP-1 stimulatory activity

**Figure 4:**
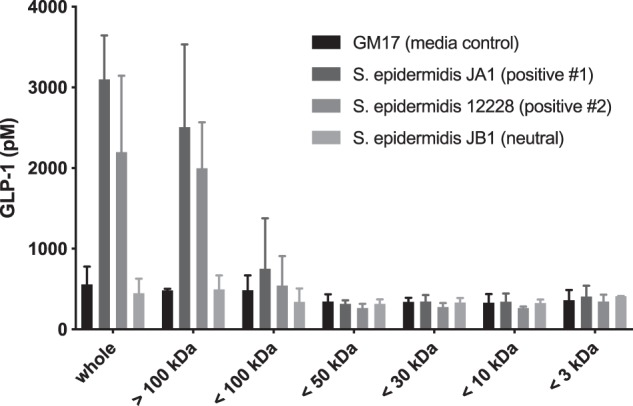
Impact of size fractionation of *S. epidermidis* supernatants on GLP-1 secretion by NCI H716 cells. Supernatants were fractionated using appropriately sized Amicon spin filters. Total GLP-1 levels were measured by ELISA (Millipore).

To identify the bacterial component responsible for GLP-1 secretion *in vitro* and for metabolic disease marker modulation *in vivo*, we performed size fractionation studies on conditioned supernatants of *S. epidermidis*. We used two GLP-1 stimulatory *S. epidermidis* strains, JA1 and the *S. epidermidis* type strain ATCC 12228, for these studies. As shown in Fig. 4, the vast majority of the GLP-1 stimulatory activity was present in the greater than 100 kDa fraction of the bacterial supernatants (2507 ± 1000 pM of GLP-1 for JA1, 1998 ± 570 pM of GLP-1 for ATCC 12228 compared to 482 ± 20 pM for the media control) with little activity remaining in the less than 100 kDa fractions (752 ± 625 pM of GLP-1 for JA1, 545.3 ± 363.5 pM of GLP-1 for ATCC 12228, compared to 487 ± 182 pM for the media control) and no activity in the less than 3 kDa fraction (410 ± 131 pM of GLP-1 for JA1, 347 ± 85 pM of GLP-1 for ATCC 12228 compared to 482 ± 20 pM for the media control). We also determined that the bacterial component is completely resistant to heat exposure (100 °C for 30 min) and Proteinase K treatment (50 µg/mL for 1 h).

To further identify the component responsible for the activity, we analyzed trypsinized bacterial supernatants by LC/MS using an Orbitrap Fusion mass spectrometer equipped with an Easy Nanospray HPLC system. Analysis of the GLP-1 stimulatory and neutral *S. epidermidis* supernatants identified 269 protein groups, of which none were detected in the GM17 medium control. A secreted peptide, with amino acid sequence MAADIISTIGDLVKWIIDTVNKFKK and a size of 3 kDa was detected in the form of two trypsin-digested peptides (Suppl. Figure 1) in the GLP-1 stimulatory *S. epidermidis* supernatants of JA1 and JA8 but absent in the GLP-1 neutral strains, JB1 and JD11. This GLP-1 stimulatory peptide was shown to have sequence homology to Hld from *Staphylococcus aureus* (denoted Hld_Sa_), a phenol soluble modulin that forms a multimeric complex in cell membranes^16^. Previous work on Hld_Sa_ has shown that it self-aggregates and was originally purified as a 270 kDa protein complex, consistent with observation of the 3 kDa peptide having an activity present in the greater than 100 kDa fraction.

**Figure 5:**
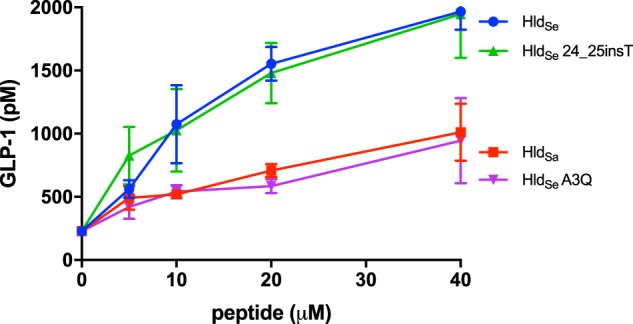
Dose dependent activity of Hld_Se_, Hld_Sa_ and two Hld_Se_ mutants, Hld_Se_ 24_25insT and Hld_Se_ A3Q, on GLP-1 secretion from NCI H716 cells. Peptides were incubated with NCI H716 cells for 2 hours after which GLP-1 levels were measured by ELISA.

Sequence alignment between *S. aureus* Hld_Sa_ and *S. epidermidis* Hld_Se_ revealed two amino acid differences between Hld_Se_ and Hld_Sa_: a substitution of alanine for glutamine at position 3 and a deletion of a threonine at position 25, yielding a 25 amino acid Hld_Se_ vs 26 amino acid Hld_Sa_. We synthesized four peptides based on the *S. epidermidis* Hld background to assess the impact of the changes compared to *S. aureus* Hld_Sa_: Hld_Se_, Hld_Sa_, Hld_Se_ A3Q, and Hld_Se_ 24_25insT. Incubation of the synthesized peptides on NCI H716 cells confirmed that Hld_Se_ possesses GLP-1 stimulatory activity (1552.0 ± 134.1 pM GLP-1 with 20 µM Hld_Se_ compared to 227.9 ± 10.0 pM GLP-1 for the media control) (Fig. 5). In addition, this activity is sequence specific as it is reduced in the *S. aureus* Hld_Sa_ (706.8 ± 52.6 pM GLP-1 with 20 µM peptide) as well as in one of the variants, Hld_Se_ A3Q (584.6 ± 56.1 pM GLP-1 with 20 µM peptide). Interestingly, the Hld_Se_ 24_25insT variant retained GLP-1 stimulatory activity (1478.8 ± 238.1 pM GLP-1 with 20 µM peptide).

### Hld_Se_ can modulate intracellular calcium levels

**Figure 6:**
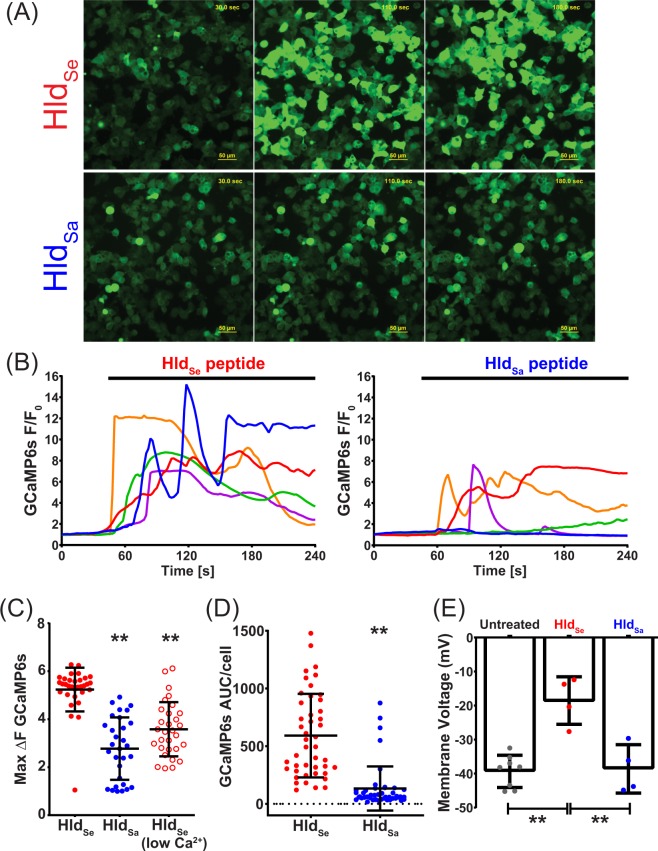
The role of Hld on calcium signalling. (**A**) Fluorescence microscopy imaging of intracellular calcium flux using HEK 293 GCaMP6S cells exposed to Hld_Se_ and Hld_Sa_. (**B**) Single cell calcium traces for five representative cells treated with Hld_Se_ (left) or Hld_Sa_ (right). GCaMP6s is shown as relative fluorescence (F/F_0_). (**C**) Quantification of the maximum increase in GCaMP6s fluorescence. **p < 0.01 by Kruskal-Wallis One-way ANOVA. (**D**) Quantification of the area under the curve (AUC) for GCaMP6s fluorescence in cells treated with Hld_Se_ or Hld_Sa_. **p < 0.01 by Mann Whitney t test. (**E**) Patch clamp of NCI H716 cells to measure membrane depolarization for untreated cells or cells treated with Hld_Se_ or Hld_Sa_ peptides (**p < 0.01 by ordinary One-way ANOVA).

We hypothesized that Hld_Se_ may be stimulating the release of GLP-1 by altering calcium signalling. To investigate this possibility, we used HEK293 cells stably expressing a genetically-encoded calcium sensor GCaMP6s, which exhibits increased green fluorescence upon an increase in cytosolic calcium levels^17^. Using the HEK293-GCaMP6S cell line, we found that treatment with 5 µM Hld_Se_ induced a strong increase in cytoplasmic calcium, as shown by the levels of green fluorescence signal following exposure to the peptide and the Hld_Se_-induced increase in calcium levels appeared to be greater and more stable than the calcium response to Hld_Sa_ (Fig. 6A). Representative single cell calcium responses to each peptide confirmed that the Hld_Se_-induced increase in cytosolic calcium had a higher magnitude and longer duration than that of the Hld_Sa_ peptide (Fig. 6B). To quantitate the magnitude of the calcium signals induced by Hld_Se_ and Hld_Sa_, we determined the maximum increase in GCaMP6s fluorescence, as well as the area under the curve (AUC) for the GCaMP6s signal upon peptide stimulation. We found that the increase in cytosolic calcium and AUC induced by Hld_Se_ was significantly greater than that induced by Hld_Sa_ (Fig. 6C,D), which is consistent with the greater GLP-1 response generated by Hld_Se_. Extracellular calcium was important for the Hld_Se_-induced increase in cytoplasmic calcium because EDTA chelation of extracellular calcium significantly reduced the calcium flux (Fig. 6C, open circles). Calcium influx through the plasma membrane would induce an increase in the membrane voltage (Vm), depolarizing the membrane potential. Thus, we performed current clamp electrophysiology studies on NCI H716 cells to measure changes in the membrane voltage upon treatment with Hld_Sa_ or Hld_Se_. Both untreated and Hld_Sa_-treated cells maintained a Vm of approximately −40 mV, but cells treated with Hld_Se_ induced a rapid and significant depolarization of NCI H716 cells (Fig. 6E). Taken together, these data indicate that Hld_Se_ may stimulate GLP-1 release via a calcium-dependent mechanism.

### The effect of Hld_Se_ on the release of other enteroendocrine cell molecules

**Figure 7:**
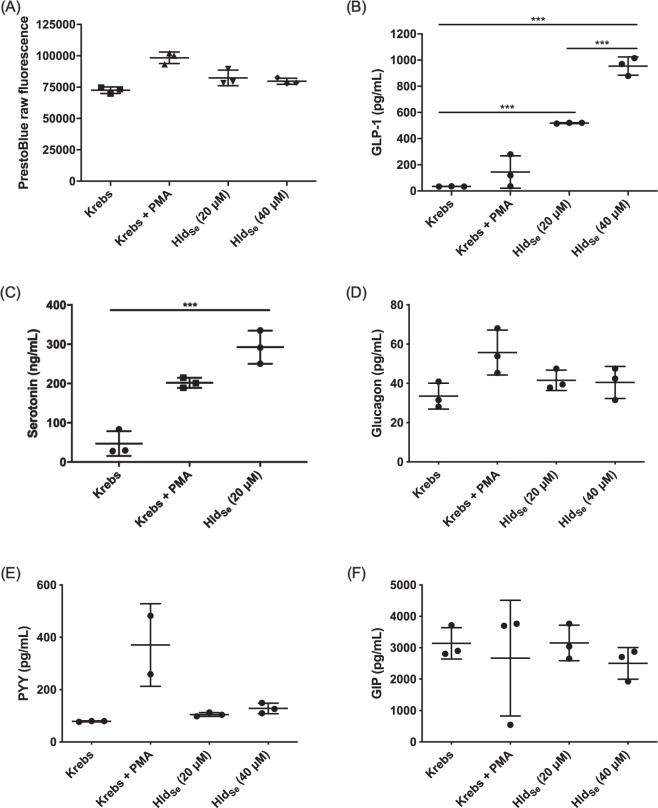
Hld_Se_ exposure on neurogenin-3 transduced human intestinal enteroids demonstrates a specificity in its activity. Hld_Se_ exposure did not lead to a loss of (**A**) cell viability as determined by a resazurin-based PrestoBlue assay. Hld_Se_ did enhance the secretion of (**B**) GLP-1 and (**C**) serotonin but not (**D**) glucagon, (**E**) Peptide-YY and (**F**) gastric inhibitory peptide (***p < 0.001, one-way ANOVA).

We further wanted to investigate the specificity of Hld_Se_ activity and its ability to stimulate the release of other enteroendocrine cell molecules. We have developed a novel human enteroid model using overexpression of Neurogenin-3 (a transcription factor that stimulates enteroendocrine cell differentiation, giving rise to higher enteroendocrine cell counts and GLP-1 levels). Using this model with Hld_Se_, there was no visible effect on cell viability, as measured by a resazurin-based assay (Fig. 7A), indicating that Hld_Se_ was not toxic to the cells. As expected, Hld_Se_ enhanced GLP-1 secretion in the enteroid model (Fig. 7B), again validating the NCI H716 and GLUTag GLP-1 data. Interestingly, Hld_Se_ exposure also stimulated the release of another gastrointestinal molecule, serotonin (Fig. 7C). Conversely, Hld_Se_ did not stimulate the release of glucagon (Fig. 7D), peptide YY (Fig. 7E), or gastric inhibitory peptide (Fig. 7F). Taken together, this data suggests specificity in the Hld_Se_ mechanism of action for the release of gastrointestinal hormones, as not all hormones were released upon Hld_Se_ exposure.

## Discussion

Regulation of intestinal hormones and physiology by microbes and microbial metabolites offers a novel approach to guide intestinal and systemic health. We targeted the incretin hormone GLP-1 due to its well-established impact on satiety and hyperglycemia, described in detail in a recent review^18^. Indeed, treatment of type 2 diabetes with GLP-1 receptor agonists, including FDA-approved exenatide and liraglutide, have demonstrated significant reductions in hyperglycemia, haemoglobin A1c, and body weight^19^. Previous studies have identified the gut microbiota as a key component in the regulation of GLP-1 secretion, although the mechanisms of action and organisms responsible for this activity have not been well-defined^20^. In this work, we initially aimed to identify human-derived microbial strains capable of promoting GLP-1 secretion. Despite extensive screening of over 1500 strains, only strains of *S. epidermidis*, isolated from either breast milk or fecal samples of healthy donors, were able to stimulate secretion of GLP-1.

*S. epidermidis* is an indigenous member of the skin microbiota, as well as the intestinal microbiota of infants in developed countries, and plays a key role for the proper education of the immune system at the skin surface^21,22^. However, much less is known about how *S. epidermidis* impacts the intestinal tract and gut function. Much of the work on *S. epidermidis* has focused on its role as a pathogen, often identified as a cause of catheter acquired infections. Thus *S. epidermidis* has traditionally been thought of as an opportunistic pathogen or a “pathobiont”. However, more recent work has suggested that *S. epidermidis*, unlike its more sinister cousin *S. aureus*, does not possess bona fide virulence factors or toxins and has instead been referred to as an “accidental pathogen”^23^.

The strongest GLP-1 stimulator strain *in vitro*, *S. epidermidis* JA1, a human breast-milk isolate, reduced weight gain and fat accumulation in HFD-fed mice over the course of a long-term 16-week study. The reduction in weight and adiposity was associated with a marked decrease in food intake, a function controlled by the action of GLP-1. In our humanized microbiota mice we did not observe a statistically significant increase in fasted glycemia between the high-fat and control diet mice and thus we could not fully assess the role of *S. epidermidis* JA1 on type 2 diabetes (although we note the *S. epidermidis* group trended toward a lower fasted glycemia, as seen in the control mice (Suppl. Figure 2)). Nevertheless, the observed resistance to HFD-induced hyperglycemia by the humanized microbiota mice that is typically observed in conventional C57BL/6 mice is of interest for further investigation. We did observe a significant change in the fasted serum insulin levels with the *S. epidermidis* group, dramatically lowering fasted insulin levels. This indicates that *S. epidermidis* treatment impacts hyperinsulinemia, a key driver of insulin resistance, metabolic syndrome, and other disorders including cardiomyopathy^24^. Unfortunately we were unsuccessful, despite many different attempts and strategies, to consistently measure serum GLP-1 levels in mice in this experiment or other positive control experiments^25^. Thus, we cannot conclude that the improved health of the animals correlates with increased levels of GLP-1 *in vivo* and will likely need to move to a larger animal model to address this question.

The identification of Hld_Se_ as the factor responsible for the secretion of GLP-1 allowed us to further investigate how this 25 amino acid peptide impacts cell physiology. Both Hld_Se_ and Hld_Sa_ belong to a class of peptides called phenol soluble modulins (PSMs). PSMs have been most extensively studied in *S. aureus* and have many possible roles in host-pathogen interactions; in particular, cytolytic properties against host red and white blood cells have been attributed to these peptides, characterizing them as virulence factors for *S. aureus*^26^. Several observations we made in these studies support that Hld_Se_ is not impacting GLP-1 secretion through its cell lysis activity. First, we observed no cell lysis nor toxicity using NCI H716, GLUTag, and human intestinal enteroids when incubated with Hld_Se_ under the conditions tested. Previous studies with Hld_Se_ and Hld_Sa_ show that lysis of erythrocytes or neutrophils occurs within 1–3 hours, which is within the timeframe or our studies^27^. Therefore, if cell lysis due to pore formation by Hld_Se_ was driving GLP-1 release we would have observed toxicity in our studies. Secondly, we found that incubation with Hld_Se_ selectively released GLP-1 and serotonin while having no impact on PYY, glucagon, or GIP. If Hld_Se_ were acting by non-selectively forming pores in the membrane we would have expected all of the hormones tested to have been increased by Hld_Se_. Finally, a previous study of Hld_Se_ on mast cell degranulation showed that under concentrations within our study (30 µM) Hld_Se_ displayed no cell lysis nor toxicity. The authors concluded that Hld_Se_ was acting by signaling directly^28^. Taken together, our data and this previous study support that Hld_Se_ can act on cell physiology independent of its non-specific pore forming activity.

Interestingly, during the initial experiments identifying *S. epidermidis* strains with the ability to stimulate the secretion of GLP-1 *in vitro*, we also identified two strains of *S. epidermidis* without stimulatory activity, and without Hld_Se_ detectable by mass spectrometry. By whole genome sequencing and comparative analysis of two GLP-1 stimulatory and two GLP-1 neutral *S. epidermidis* strains, we demonstrated that the *hld* gene is present and homologous in all of the sequenced *S. epidermidis* strains, regardless of GLP-1 stimulatory activity. However, we identified a single nucleotide polymorphism (SNP) (Suppl. Figure 3) in the *agrA* gene of the neutral strains. AgrA is part of an autoregulatory quorum-sensing system that controls the expression of Hld_Se_. In *S. aureus*, a mutation in *agrA* leads to a loss in haemolytic activity^29^, and we hypothesize that the identified SNP in the GLP-1 neutral *S. epidermidis* strains accounts for the absence of Hld_Se_ production and thus, GLP-1 stimulatory activity. Naturally occurring *agr* mutants of *S. epidermidis* and *S. aureus* have been previously described^30,31^.

*S. epidermidis* is an interesting conundrum for microbiome researchers: despite assumed danger, it clearly presents as a mutualistic organism that provides health benefits for the host, including immune regulation and barrier defense to skin pathogens. Indeed, Hld_Se_ has been directly linked to enhancing the properties of the defensin LL-37 against the skin pathogen Group A *Streptococcus*^22^. However, most of the research conducted on the species has involved its role in pathogenesis, not as a microbial therapeutic. Although the research community should consider the use of non-traditional microbial strains as therapeutics, with proper safety characterization of course, future studies may aim to develop Hld_Se_ as a peptide-therapeutic independent of *S. epidermidis*. Added microbial therapeutic potential may exist in the expression of Hld_Se_ in an organism like *Lactobacillus reuteri*, which already has inherent therapeutic properties, has Generally Recognized as Safe status, and is suitable for delivery to the gastrointestinal tract.

## Supplementary information

Supplementary information.

## Data Availability

All data and reagents will be made available upon request.
