# Supplementary material for: Discovery of a bacterial peptide as a modulator of GLP-1 and metabolic disease
Source: Sci Rep. 2020 Mar 18;10:4922. doi: 10.1038/s41598-020-61112-0 (PMC7080827; doi:10.1038/s41598-020-61112-0)
Supplement: Supplementary file 1 — Supplementary information. [file 41598_2020_61112_MOESM1_ESM.pdf]

Supplementary Material for the manuscript:

Discovery of a bacterial peptide as a modulator of GLP-1 and metabolic disease

Running title: Novel *S. epidermidis* peptide modulates GLP-1 levels

Catherine Tomaro-Duchesneau[1], Stephanie L. LeValley[1], Daniel Roeth[2], Liang Sun[3], Frank T. Horrigan[3], Markus Kalkum[2], Joseph M. Hyser[1], Robert A. Britton[1*]

A

MS/MS of M(ox)AADIISTIGDLVK

731.90 m/z, 2+, 1,461.78 Da, (Parent Error: 0.21 ppm)

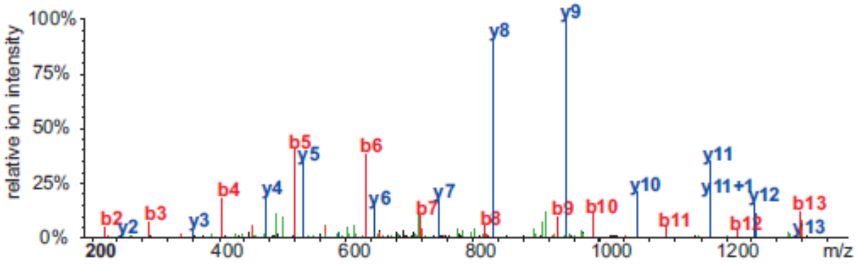

B	b ions	b^{2+}	b-H_2O	AA	y ions	y^{2+}	y-NH_3	y-H_2O	Y
1	148.0			M(ox)	1,462.8	731.9	1,445.8	1,444.8	14
2	219.1			A	1,315.8	658.4	1,298.7	1,297.7	13
3	290.1			A	1,244.7	622.9	1,227.7	1,226.7	12
4	405.1		387.1	D	1,173.7	587.3	1,156.7	1,155.7	11
5	518.2		500.2	I	1,058.7	529.8	1,041.6	1,040.6	10
6	631.3	316.2	613.3	I	945.6	473.3	928.5	927.6	9
7	718.3	359.7	700.3	S	832.5	416.7	815.5	814.5	8
8	819.4	410.2	801.4	T	745.5	373.2	728.4	727.4	7
9	932.5	466.7	914.5	I	644.4	322.7	627.4	626.4	6
10	989.5	495.3	971.5	G	531.3		514.3	513.3	5
11	1104.5	552.8	1086.5	D	474.3		457.3	456.3	4
12	1217.6	609.3	1199.6	L	359.3		342.2		3
13	1316.7	658.8	1298.7	V	246.2		229.2		2
14	1462.8	731.9	1444.8	K	147.1		130.1		1

B

MS/MS of WIIDTVNK

494.78 m/z, 2+, 987.54 Da, (Parent Error: 0.83 ppm)

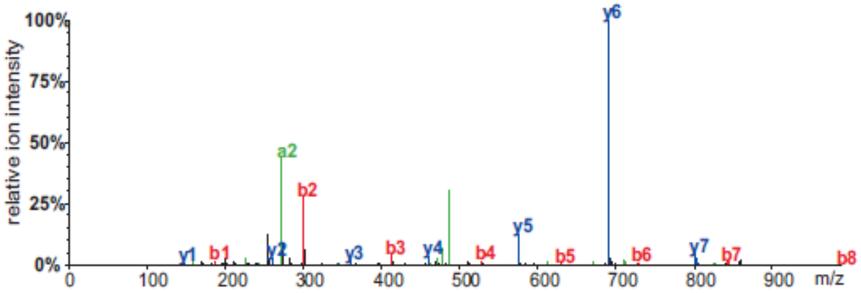

B	b ions	b^{2+}	b-H_2O	AA	y ions	y^{2+}	y-NH_3	y-H_2O	Y
1	187.1			W	988.6	494.8	971.5	970.5	8
2	300.2			I	802.5	401.7	785.4	784.5	7
3	413.3			I	689.4	345.2	672.4	671.4	6
4	528.3		510.3	D	576.3		559.3	558.3	5
5	629.3		611.3	T	461.3		444.3	443.3	4
6	728.4	364.7	710.4	V	360.2		343.2		3
7	842.4	421.7	824.4	N	261.2		244.1		2
8	988.6	494.8	970.5	K	147.1		130.1		1

Suppl Fig 1: Two trypsin-derived Hld peptides, M(ox)AADIISTIGDLVK and WIIDTVNK with mass over charge (m/z) ratios of 731.90 and 494.78 were identified, which resulted in a 88% coverage of Hld_{Se} (MAADIISTIGDLVKWIIDTVNKFKK). MS/MS spectra and fragmentation tables of (A) the peptide M(ox)AADIISTIGDLVK with an oxidized methionine at the first position and (B) the peptide WIIDTVNK.

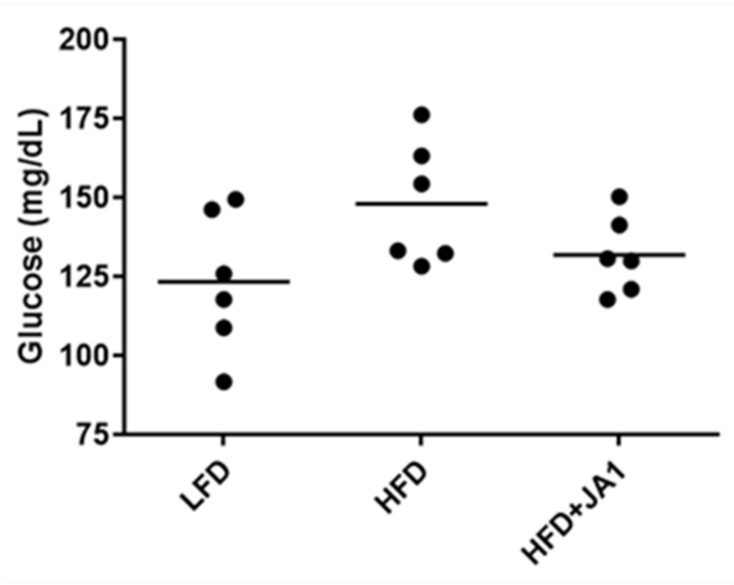

Suppl Fig 2: Effect of administration of *S. epidermidis* JA1 on fasted serum glucose at the end of a 16 week study.

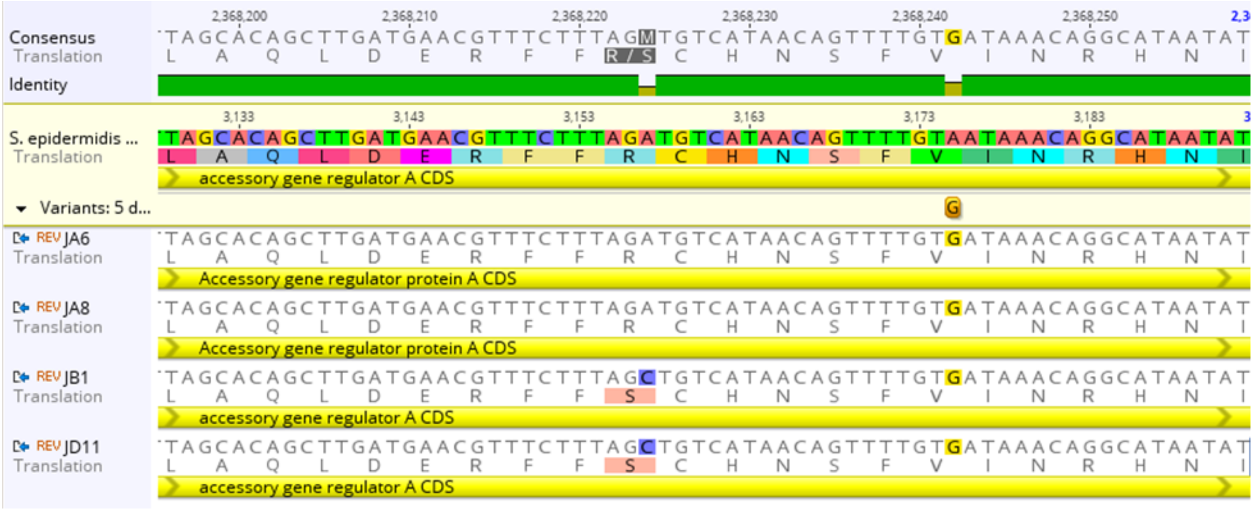

Suppl Fig 3: Identification of a SNP in accessory gene regulator A (*agrA*) in the GLP-1 neutral (JB1 and JD11) vs. the GLP-1 stimulatory (JA6 and JA8) *S. epidermidis* strains.
